# Cdk5rap3 is essential for intestinal Paneth cell development and maintenance

**DOI:** 10.1038/s41419-021-03401-8

**Published:** 2021-01-27

**Authors:** Michaela Quintero, Siyang Liu, Yanhua Xia, Yonghong Huang, Yi Zou, Ge Li, Ling Hu, Nagendra Singh, Richard Blumberg, Yafei Cai, Hong Xu, Honglin Li

**Affiliations:** 1 Department of Biochemistry & Molecular Biology, Medical College of Georgia, Augusta University, Augusta, GA 30912 USA; 2 Faculty of Basic Medicine, Nanchang University, Nanchang, Jiangxi China; 3 Department of Metabolic Endocrinology, The Third Affiliated Hospital of Nanchang University, Nanchang, Jiangxi China; 4 Department of Pathology, Sir Run Run Shaw Hospital, Zhejiang University, Hangzhou, China; 5 Division of Gastroenterology, Department of Medicine, Brigham and Women’s Hospital, Harvard Medical School, Boston, MA 02115 USA; 6 College of Animal Science and Technology, Nanjing Agricultural University, Nanjing, Jiangsu China

**Keywords:** Ubiquitylation, Differentiation

## Abstract

Intestinal Paneth cells are professional exocrine cells that play crucial roles in maintenance of homeostatic microbiome, modulation of mucosal immunity, and support for stem cell self-renewal. Dysfunction of these cells may lead to the pathogenesis of human diseases such as inflammatory bowel disease (IBD). Cdk5 activator binding protein Cdk5rap3 (also known as C53 and LZAP) was originally identified as a binding protein of Cdk5 activator p35. Although previous studies have indicated its involvement in a wide range of signaling pathways, the physiological function of Cdk5rap3 remains largely undefined. In this study, we found that Cdk5rap3 deficiency resulted in very early embryonic lethality, indicating its indispensable role in embryogenesis. To further investigate its function in the adult tissues and organs, we generated intestinal epithelial cell (IEC)-specific knockout mouse model to examine its role in intestinal development and tissue homeostasis. IEC-specific deletion of *Cdk5rap3* led to nearly complete loss of Paneth cells and increased susceptibility to experimentally induced colitis. Interestingly, *Cdk5rap3* deficiency resulted in downregulation of key transcription factors Gfi1 and Sox9, indicating its crucial role in Paneth cell fate specification. Furthermore, Cdk5rap3 is highly expressed in mature Paneth cells. Paneth cell-specific knockout of *Cdk5rap3* caused partial loss of Paneth cells, while inducible acute deletion of Cdk5rap3 resulted in disassembly of the rough endoplasmic reticulum (RER) and abnormal zymogen granules in the mature Paneth cells, as well as loss of Paneth cells. Together, our results provide definitive evidence for the essential role of Cdk5rap3 in Paneth cell development and maintenance.

## Introduction

The intestinal epithelium consists of a single layer of epithelial cells, and its primary function includes absorbing nutrients and serving as a barrier against luminal pathogens. To maintain the intestinal integrity, intestinal stem cells (ISCs), located in the bases of crypts of Lieberkuhn, rapidly proliferate and differentiate into mature epithelial cells. Among major types of intestinal epithelial cells (IECs), absorptive enterocytes are mainly responsible for nutrient absorption, whereas professional secretory cells such as Paneth and goblet cells play crucial roles in maintaining intestinal homeostasis and mucosal immunity. Interspersed with ISCs at the base of crypts, Paneth cells are distinctive professional exocrine cells containing an extensive rough endoplasmic reticulum (RER) network and many zymogen granules. They synthesize and secrete a large quantity of antimicrobial peptides/proteins and inflammatory cytokines that are essential for shaping healthy gut microbiome and maintaining innate immunity^1^. Loss or impairment of Paneth cell function is often observed in the patients with inflammatory bowel disease, and contributes to the onset and progression of the disease^2–4^. Paneth cells also produce niche factors EGF, Wnt3, and Notch ligands to support Lgr5^+^ intestinal stem cells^5^. Moreover, Paneth cells can serve as key nutrient sensors to enhance stem cell self-renewal in response to calorie restriction, further highlighting their role as ISC niche to couple organismal nutritional status to stem cell function^6^. More recently, several studies have demonstrated that Paneth cells possess multipotency to transdifferentiate into other types of IECs and directly contribute to intestinal regeneration under stress conditions^7–9^. Therefore, elucidation of the molecular and cellular mechanisms that regulate Paneth cell development, function, and plasticity would be crucial for understanding of intestinal homeostasis and disease pathogenesis.

Cdk5 activator binding protein Cdk5rap3 (also known as C53 and LZAP) was originally identified as a binding protein of Cdk5 activator p35, CBP, and ARF protein^10–12^. It is highly conserved in multi-cellular organisms, and its orthologues are found in vertebrate, invertebrate and plants but not in yeast and bacteria. Previous studies indicate its involvement in a wide variety of signaling pathways, including NF-κB^13^, ARF/p53^11,14^, Wnt^15–17^, STAT3^18^, DNA damage response^19,20^ and UFMylation^21,22^. Not surprisingly, it was reported to interact with various proteins, including p35^10^, CBP/p300^12^, Rel A^13^, Chk1/2^20^, PAK4^23^, ARF^11^, p38MAPK^24^, UFL1 (also known as RCAD, NLBP and Maxer)^25–27^, γ-tubulin^28^, and TIP-1^29^. Clinical studies using cancer patient samples have suggested its tumor suppressing activity in head/neck and gastric cancers^13,14,16,17,30^, but the conflicting results were reported in liver cancer^23,31^. Despite its potential involvement in many important signaling pathways, Cdk5rap3’s physiological functions remain poorly defined. In a zebrafish model, Liu et al. have shown that Cdk5rap3 is essential for early zebrafish development^32^. More recenlty, Yang et al. reported that *Cdk5rap3* deficiency in mice led to embyonic lethality possibly due to severe liver hypoplasia^22^. Hepatocyte-specific *Cdk5rap3* knockout mice suffered post-weaning lethality, owing to serious hypoglycemia and impaired lipid metabolism^22^. These findings have demonstrated an essential role of Cdk5rap3 in both embryogenesis and organ development.

In this study, we found that Cdk5rap3 is highly expressed in intestinal Paneth cells. IEC-specific knockout of Cdk5rap3 led to nearly complete absence of Paneth cells and increased susceptibility to experimentally induced colitis. *Cdk5rap3* deficiency impaired the development of intestinal stem cells into Paneth cell lineage. Furthermore, Paneth cell-specific deletion of *Cdk5rap3* caused partial loss of Paneth cells, while its acute ablation resulted in disassembly of RER, abnormality of zymogen granules, and loss of mature Paneth cells. Taken together, our results clearly demonstrate that Cdk5rap3 is required in Paneth cell development and maintenance.

## Results

### *Cdk5rap3* knockout leads to early embryonic lethality

To elucidate the physiological function of Cdk5rap3, we generated *Cdk5rap3* knockout mice using ES cell clone from the Knockout Mice Project (KOMP). Insertion of the “lacZ-neo” (>20 kb) cassette between exons 5 and 6 disrupted normal RNA splicing, resulting in a knockout allele and a truncated protein product (we designate this allele as “m”) (Supplementary Fig. S1a, b). Crossing of heterozygous mice failed to produce the pups with homozygous knockout alleles, suggesting that *Cdk5rap3* deficiency causes embryonic lethality (Supplementary Fig. 1c). Indeed, no *Cdk5rap3* deficient embryos were recovered from timed pregnant mice after E8.5, a result that seems to be consistent with the report by Liu et al.^32^.

### IEC-specific deletion of *Cdk5rap3* leads to complete depletion of Paneth cells

**Fig. 1:**
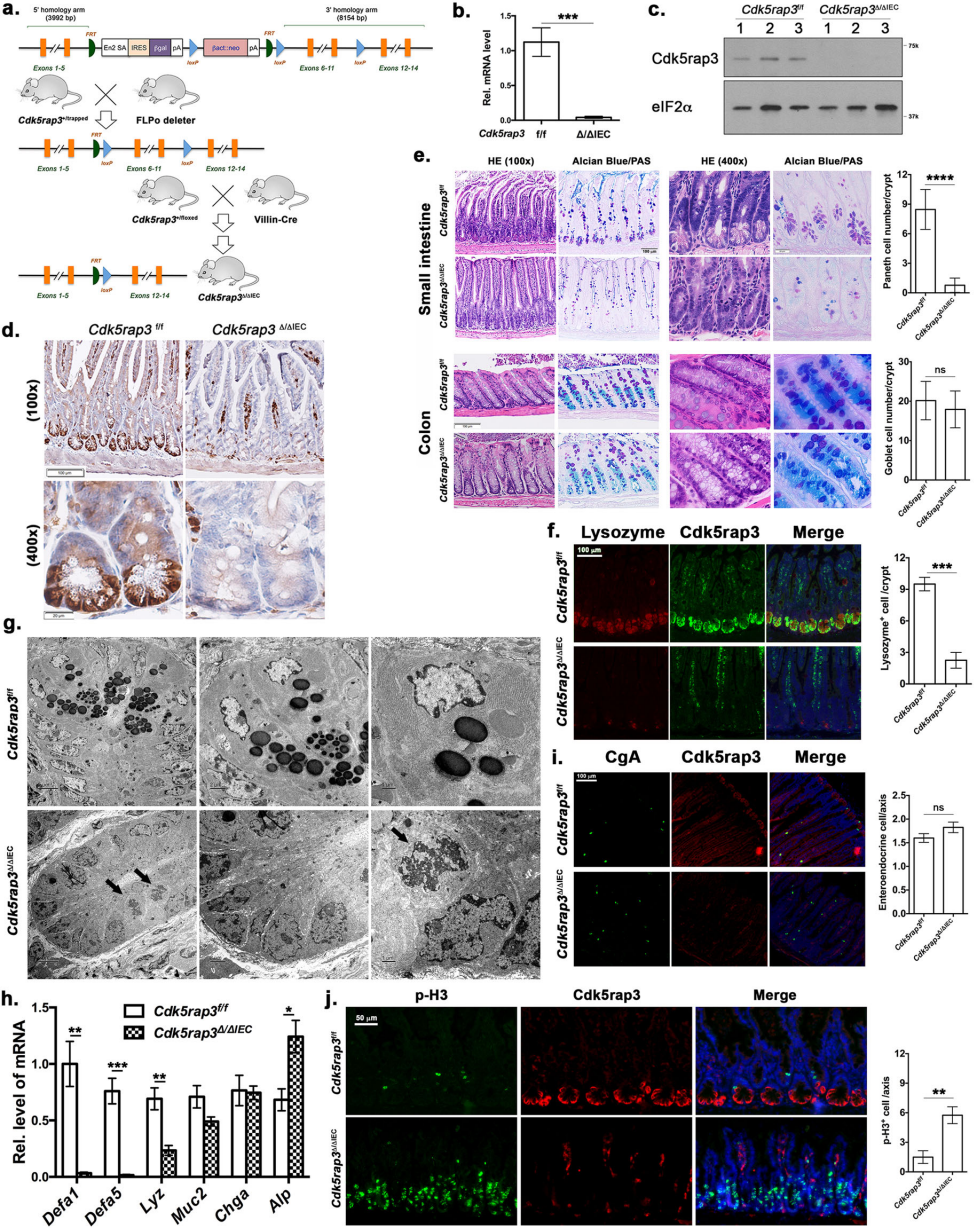
Intestinal epithelial cell (IEC)-specific deletion of Cdk5rap3 leads to ablation of Paneth cells. **a** Scheme of mouse breeding to generate IEC-specific KO mice. **b** Quantitative RT-PCR analysis of *Cdk5rap3* mRNA using the primers specific for floxed exons (*n* = 3 mice per genotype); Total RNA was isolated from intestinal crypts. **c** Immunoblotting of Cdk5rap3 in the lysate of intestinal epithelial cell lysates. **d** Immunohistochemistry of Cdk5rap3 in wild-type and *Cdk5rap3* KO ileal sections. **e** Significant loss of Paneth cells in *Cdkrap3*^∆/∆IEC^ intestine. The number of Paneth and goblet cells were counted in double-blinded fashion from more than 20 crypts, and 4 mice of each phenotypes were scored. ****p < 0.0001 (*n* = 4 mice per genotype). **f** Lysozyme staining of ileal sections of wild-type and *Cdkrap3*^∆/∆IEC^ mice. Lysozyme-positive cells per crypt were scored. ***p < 0.001 (*n* = 4 mice per genotype). **g** Electron micrographs of the crypts of wild-type and *Cdk5rap3*^∆/∆IEC^ mice. Mitotic cells with condensed chromatin were marked by solid arrows. **h** Quantitative RT-PCR analysis of cell type-specific gene expression. Total RNA was isolated from scraped cells of ileal section. *p < 0.05, **p < 0.01, ***p < 0.001 (*n* = 4 mice per genotype). **i** The number of enteroendocrine cells (Chromogranin A-positive) in wild-type and *Cdkrap3*^∆/∆IEC^ intestine. The number of ChA^+^ cells per crypt-villus axis was counted and four mice of each phenotypes were scored. **j** Mitotic cells (phospho-Histone H3 Ser10 staining) in wild-type and *Cdk5rap3*^∆/∆IEC^ ileal sections. The number of p-H3^+^ cells per crypt-villus axis was scored. **p < 0.01. (*n* = 4 mice per genotype).

To further investigate Cdk5rap3’s function in the adult animal, we first crossed *Cdk5rap3*^+/m^ mice with FLPo deletor mice to remove the “lacZ-neo” cassette and create floxed *Cdk5rap3* allele. Subsequently, we crossed *Cdk5rap3* floxed mice with CAG-CreERT2 transgenic mice to generate the whole-body conditional KO mice. Upon tamoxifen (TAM) administration, *Cdk5rap3* deficient mice were viable, but bleeding in the gut was frequently observed. To explore the impact on the intestinal epithelium, we crossed *Cdk5rap3* floxed mice with Villin-Cre transgenic mice to generate IEC-specific knockout mice (Fig. 1a). Deficiency of *Cdk5rap3* in the intestinal epithelium was confirmed by quantitative RT-PCR (Fig. 1b), immunoblotting (Fig. 1c), and immunohistochemistry (Fig. 1d). Interestingly, we found that Cdk5rap3 was highly expressed in Paneth cells at the bottom of intestinal crypts (Fig. 1d).

IEC-specific *Cdk5rap3* KO (referred to as *Cdk5rap3*^∆/∆IEC^ hereafter) mice were born healthy without obvious developmental defects, growth retardation, and comprised gross epithelial organization (Fig. 1e). However, close examination of intestinal sections revealed substantial changes in the crypts. As shown by Alcian blue (AB)/Periodic acid–Schiff (PAS) staining, *Cdk5rap3*^∆/∆IEC^ mice almost completely lacked Paneth cells at the bottom of the crypts in the small intestine (Fig. 1e). This phenotype was further confirmed by lysozyme staining (Paneth cell marker, Fig. 1f), transmission electron microscopy (TEM) of the crypts (Fig. 1g), and RT-PCR showing significant reduction of Paneth cell-specific gene expression (defensin 1, 5 and lysozyme) (Fig. 1h). In contrast to Paneth cells, the number of goblet cells in both small and large intestine was not significantly altered even though AB/PAS^+^ granules appeared to be smaller in the small intestine of *Cdk5rap3* KO mice (Fig. 1e). In addition, *Cdk5rap3* deficiency led to a slight increase in the number of enteroendocrine cells that failed to reach statistical significance (Chromogranin A positive cells) (Fig. 1i). Overall organization of enterocytes was not affected in *Cdk5rap3*^∆/∆IEC^ mice, yet expression of enterocyte specific intestinal alkaline phosphatase was increased in *Cdk5rap3*^∆/∆IEC^ intestine (Fig. 1h). Interestingly, the cells with condensed chromatin were frequently observed in *Cdk5rap3*^∆/∆IEC^ crypts (marked by solid arrows), while such cells were rarely found in wild-type mice (Fig. 1g). These cells appeared to be in mitotic phase because more phospho-Histone H3 positive cells were observed in *Cdk5rap3*^∆/∆IEC^ crypts than wild-type (Fig. 1j). A similar phenomenon was observed during development of *Cdk5rap3* knockdown zebrafish embryos^32^.

We also took advantage of organoid culture to confirm the findings from the knockout mouse model. Interestingly, unlike *Math1* deficient crypts without Paneth cells that could not grow ex vivo organoids in the absence of exogenous Wnt ligand^33^, *Cdk5rap3* deficient crypts were able to proliferate and differentiate into well-formed organoids without exogenous Wnt ligand (Supplementary Fig. S2a). This is reminiscent of recently reported *Lsd1* deficient organoid culture^34^. *Cdk5rap3* deficient organoids did not contain Paneth cells (Supplementary Fig. S2b), and the absence of Paneth cells was further confirmed by lysozyme staining (Supplementary Fig. S2c) and RT-PCR analysis (Supplementary Fig. S2d). Therefore, the organoid culture of *Cdk5rap3* deficient crypts truthfully recapitulated the phenotype of *Cdk5rap3*^∆/∆IEC^ intestine. Collectively, our results strongly suggest that Cdk5rap3 is essential for Paneth cell development.

### *Cdk5rap3* ablation impairs fate specification of Paneth cell lineage

**Fig. 2:**
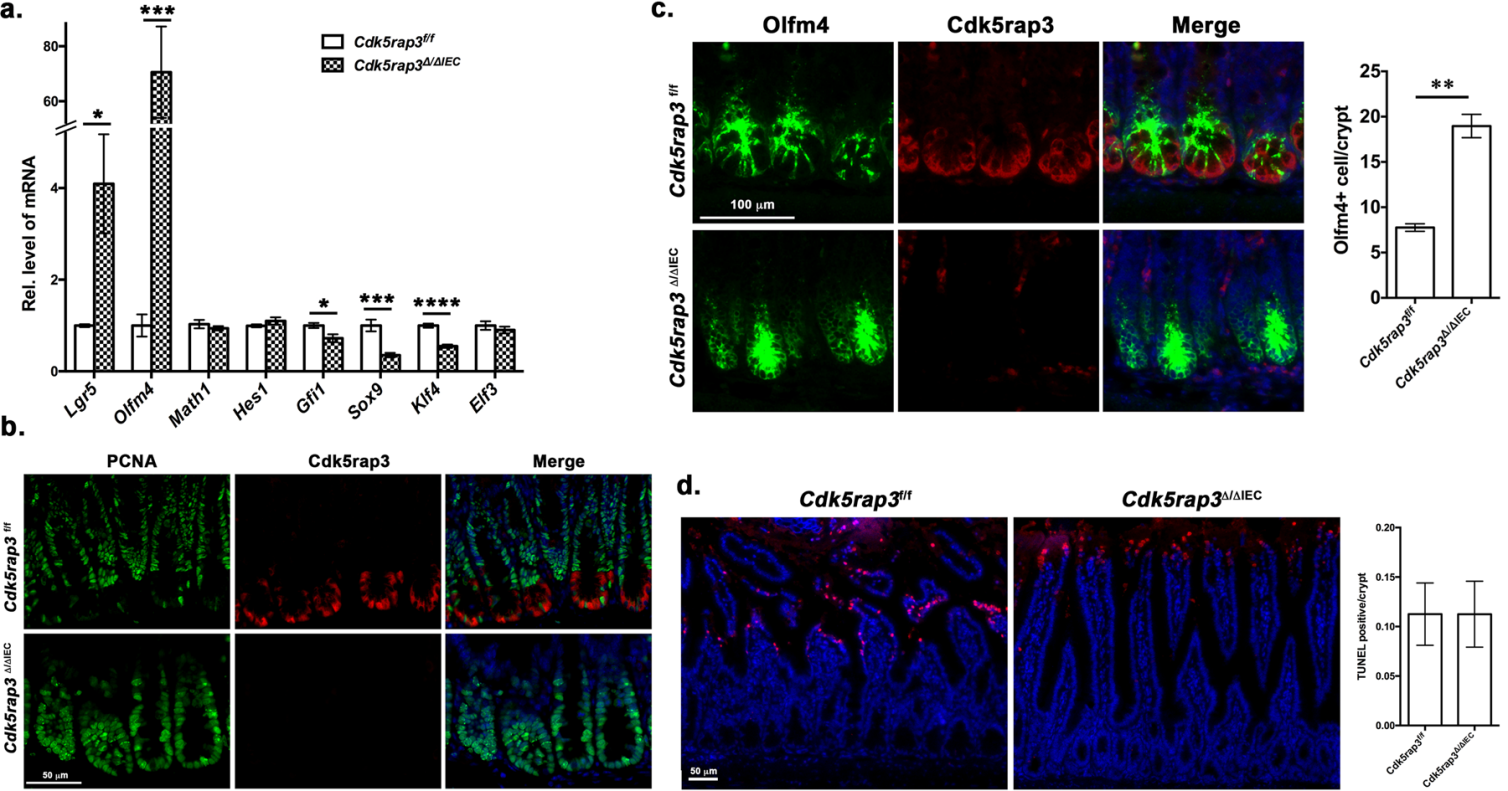
Cdk5rap3 deletion impairs fate specification of Paneth cell lineage. **a** Quantitative RT-PCR analysis of specific genes involved in intestinal stem cell renewal and differentiation. Total RNA was isolated from intestinal scrapes and used for qRT-PCR analysis. *p < 0.05, **p < 0.01, ***p < 0.001, ****p < 0.0001 (*n* = 4 mice per genotype). **b** Proliferating cells at the crypt base of wild-type and *Cdk5rap3* deficient small intestine. Ileal sections were so-stained with PCNA and Cdk5rap3 antibodies. **c** Olfm4 staining of wild-type and *Cdk5rap3*^∆/∆IEC^ ileal sections. Olfm4^+^ cells per crypt were scored and compared. **p < 0.01. (*n* = 4 mice per genotype). **d** TUNEL staining of wild-type and *Cdk5rap3*^∆/∆IEC^ ileal sections. TUNEL positive cells per crypt were scored from four mice of each genotype.

Paneth cells are derived from Math1/Atoh1 positive secretory progenitor cells, and their fate is determined by transcription factors (TFs) such as Gfi1 and Sox9^35–39^. After lineage commitment, progenitor cells further develop into the mature Paneth cells that contain extensive rough ER network and zymogen granules. The Notch-Hes1 signaling represses Math1/Atoh1 and promotes absorptive enterocyte fate^40–43^. Transcription factor Elf3 is required for differentiation of enterocytes^44^, while Klf4, along with Elf3, is essential for terminal differentiation of goblet cells^45^. We examined whether Cdk5rap3 is involved in stem cell differentiation and Paneth cell specification. As showing Fig. 2a, both Gfi1 and Sox9, two critical TFs for Paneth cell lineage, were significantly down-regulated by *Cdk5rap3* deficiency. In contrast, expression of *Hes1* and *Elf3* that are important for enterocyte development were not significantly altered by *Cdk5rap3* ablation (Fig. 2a). Expression of *Klf4* was also significantly reduced in *Cdk5rap3*^∆/∆IEC^ mice, and this reduction may contribute to smaller goblet cells observed in *Cdk5rap3*^∆/∆IEC^ small intestine (Fig. 1e). The result of TF expression profile suggests that Cdk5rap3 is critical for fate determination, and its deficiency impairs Paneth cell lineage specification. Interestingly, expression of *Lgr5* and *Olfm4*, two intestinal stem cell markers, were also dramatically elevated in *Cdk5rap3* KO intestine (Fig. 2a). While proliferating (PCNA^+^) intestinal stem cells (Olfm4^+^ cells) were located at the bottom of crypts and interspaced between Paneth cells in the wild-type mice, the crypt bases of *Cdk5rap3*^∆/∆IEC^ intestine were occupied by proliferating (PCNA^+^) and Olfm4^+^ cells (Fig. 2b, c). Further study will determine whether *Cdk5rap3* deficiency promotes ISC proliferation in a cell autonomous manner. We also examined cell death in *Cdk5rap3*^∆/∆IEC^ small intestine and found no significant difference in the number of TUNEL positive cells (Fig. 2d).

### Deletion of *Cdk5rap3* in mature Paneth cells causes their loss and abnormality

**Fig. 3:**
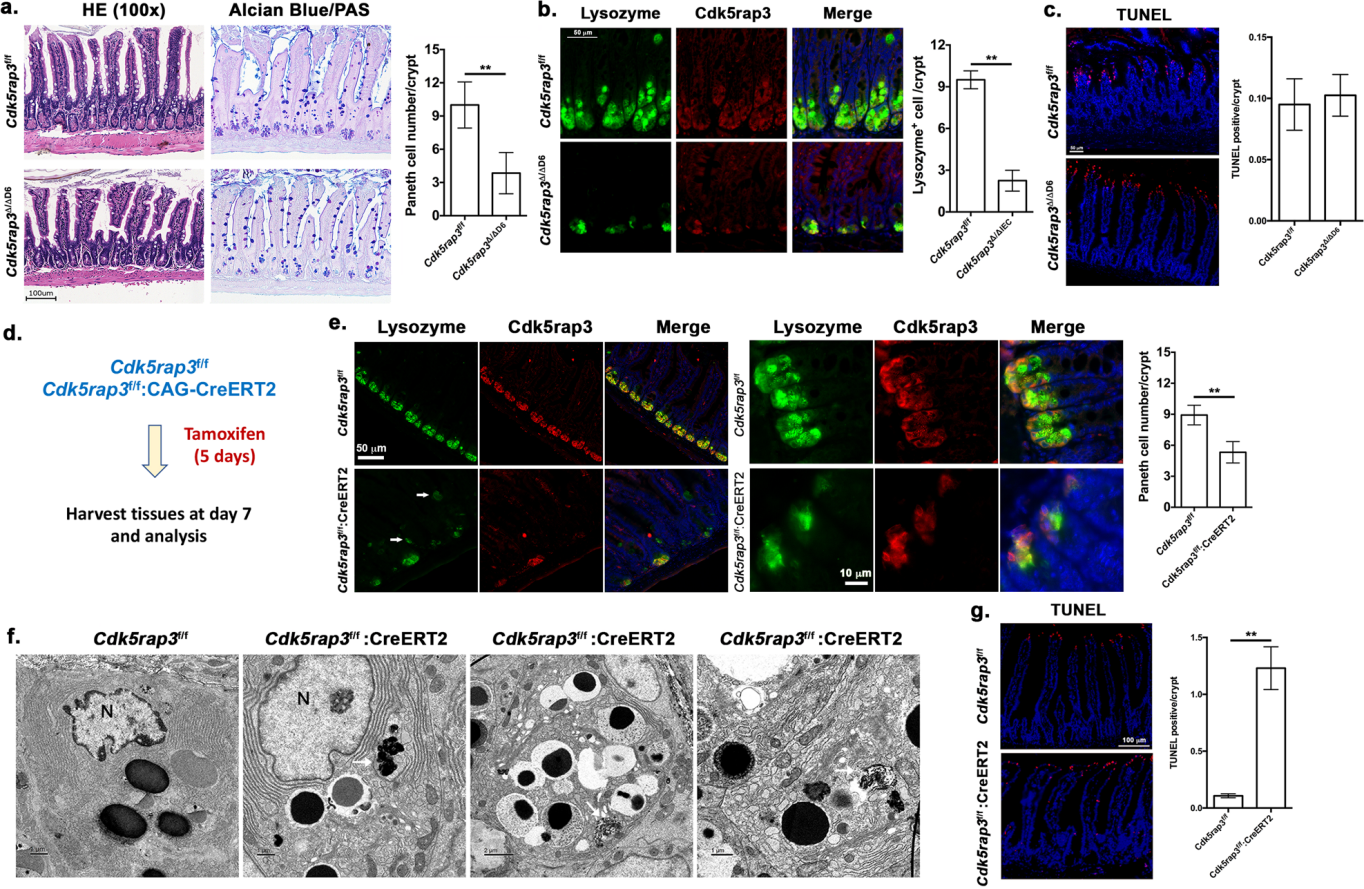
Paneth cell-specific deletion of Cdk5rap3 causes loss of Paneth cell. **a** H&E and Alcian blue/PAS staining of ileal sections of wild-type and Cdk5rap3 Paneth cell-specific *Cdk5rap3* KO mice (*Cdk5rap3*^∆/∆D6^). The number of Paneth cells per crypt was scored. **p < 0.01. (*n* = 4 mice per genotype). **b** Lysozyme staining of ileal sections of wild-type and *Cdkrap3*^∆/∆D6^ mice. Lysozyme-positive cells per crypt were scored. **p < 0.01. (*n* = 4 mice per genotype). **c** TUNEL staining of ileal sections of wild-type and *Cdkrap3*^∆/∆D6^ mice. TUNEL positive cells per crypt were scored (*n* = 4 mice per genotype). **d** Experimental procedure to analyze the effect of acute deletion of Cdk5rap3. Mice were treated with daily i.p. injection of tamoxifen (75 mg/kg) for 5 consecutive days, and the tissues were harvested at day 7 for fixation and analysis. **e** Lysozyme staining of ileal sections of wild-type and *Cdkrap3* deficient mice. Lysozyme-positive cells per crypt were scored. **p < 0.01. (*n* = 4 mice per genotype). **f** TEM analysis of the crypts of wild-type and *Cdkrap3* deficient small intestine. The nucleus was labeled as “N”, and autolysosomes were marked by white arrows. **g** TUNEL staining of ileal sections of wild-type and *Cdkrap3* deficient mice. TUNEL positive cells per crypt were scored. **p < 0.01. (*n* = 4 mice per genotype).

As shown in Fig. 1d, Cdk5rap3 is highly expressed in Paneth cells, indicating its potential role in maintenance of mature cells. To address this possibility, we generated Paneth cell-specific KO model of Cdk5rap3 using Defensin 6-Cre (D6-Cre) transgenic line^2^. *Cdk5rap3* deletion (*Cdk5rap3*^∆/∆D6^) led to significant loss of Paneth cells (Fig. 3a, b) even though the reduction was not as dramatic as in *Cdk5rap3*^∆/∆IEC^ mice (Fig. 1e). This may be caused by variable penetrance of Cre transgene expression and efficiency of Cre-mediated deletion of *Cdk5rap3* allele because most Lysozyme^+^ cells in *Cdk5rap3*^∆/∆D6^ intestine were also Cdk5rap3^+^ (Fig. 3b). We also examined cell death, and no significant increase of TUNEL-positive cells was detected in the *Cdk5rap3*^∆/∆D6^ crypts (Fig. 3c). The result of this KO model suggests that Cdk5rap3 is also important for mature Paneth cells. Further investigation using the mice with tracing reporter will determine whether *Cdk5rap3* deficient Paneth cells undergo cell death or trans-differentiate to other types of cells.

We also examined the effect of acute deletion of *Cdk5rap3* by treating *Cdk5rap3*^f/f^:CAG-CreERT2 mice with TAM. The mice were treated with TAM for 5 days and analyzed at day 7 after the first injection (Fig. 3d). TAM-induced acute deletion of *Cdk5rap3* resulted in fewer lysozyme^+^ Paneth cells, and more metaplastic Paneth cells outside of the crypts (Fig. 3e, marked by white arrows), while zymogen granules appeared to be disorganized (Fig. 3e). TEM analysis showed that *Cdk5rap3* abolition led to local disassembly of the rough ER network, increase of autophagosomes and deformed zymogen granules, a phenotype that is similar to the one of *Mist1* deficient Paneth cells (Fig. 3f)^46^. Compared to *Cdk5rap3*^f/f^ mice, the number of TUNEL-positive cells was significantly elevated in TAM-treated *Cdk5rap3*^f/f^:CAG-CreERT2 intestine (Fig. 3g). Our results suggest that Cdk5rap3 is critical for either Paneth cell maturation or maintenance of mature Paneth cells.

### *Cdk5rap3*^∆/∆IEC^ mice are more susceptible to dextran sulfate sodium (DSS)-induced colitis

**Fig. 4:**
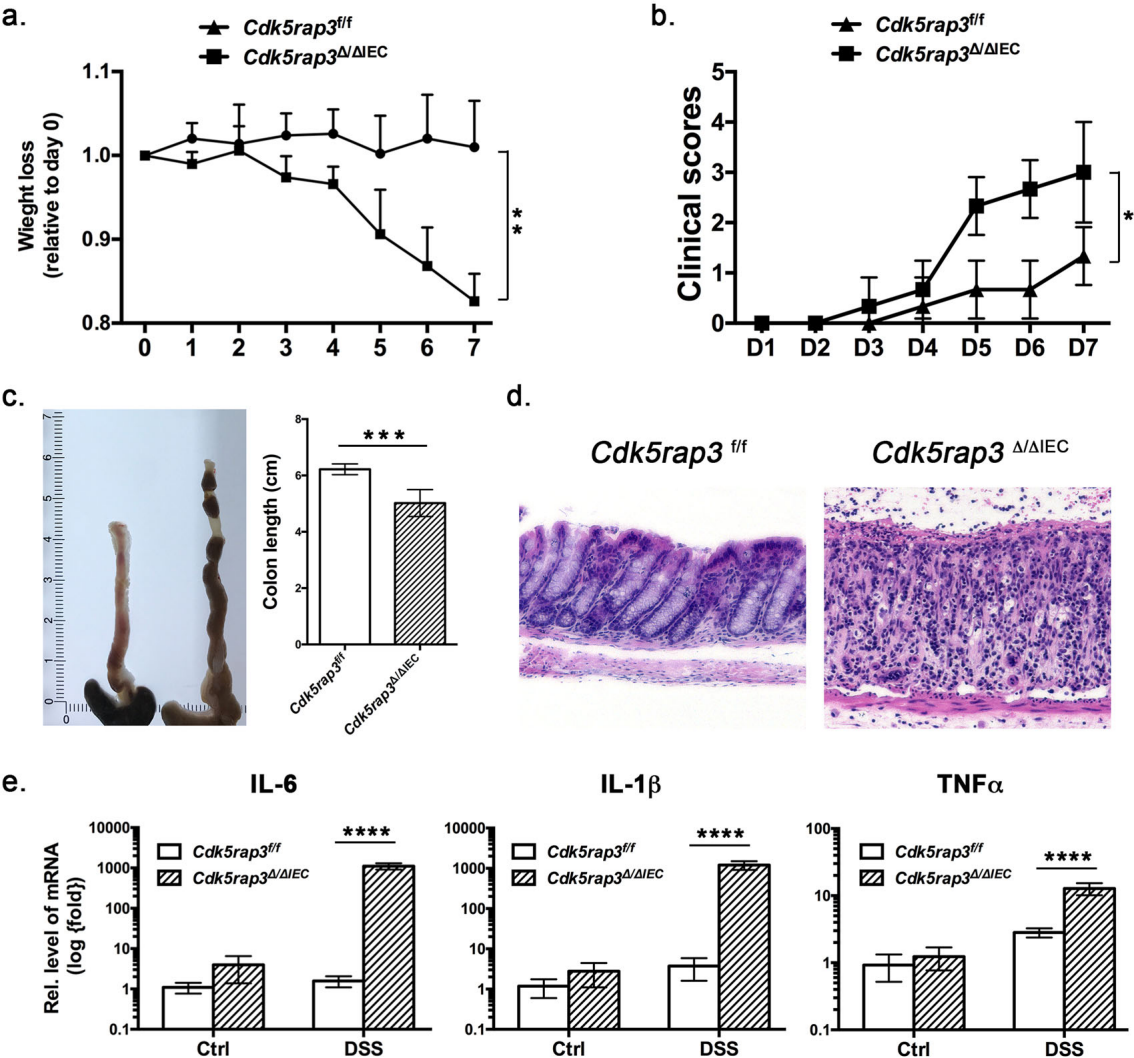
*Cdk5rap3*^∆/∆IEC^ mice are susceptible to experimentally induced colitis. Mice of each genotype were treated with 2.5% DSS in drinking water for 5 consecutive days and monitored for weight loss and clinical symptoms such as diarrhea and blood in stools. **a** Weight loss of DSS-treated mice **p < 0.01 (*n* = 5 mice per genotype). **b** Clinical scores of diarrhea of DSS-treated mice. Diarrhea (0 = no diarrhea, 1 = soft stool, 2 = very soft stool, 3 = liquid stool, 4 = severe diarrhea). *p < 0.05 (*n* = 5 mice per genotype). **c** Colon length. ***p < 0.001 (*n* = 5 mice per genotype). **d** H&E staining of colonic sections of wild-type and *Cdk5rap3* deficient mice after DSS treatment. **e** Elevated expression of inflammatory cytokines in *Ufbp1* deficient mice after DSS treatment. Total colonic RNA was subjected to quantitative RT-PCR analysis. ****p < 0.0001 (*n* = 5 mice per genotype).

Paneth cells play a pivotal role in maintaining homeostasis of intestinal microbiota and innate immunity, and impairment or loss of their function may lead to dysbiosis and inflammatory response. It has been shown that dysfunction of Paneth cells in mice leads to dysbiotic microbiota and loss of intestinal homeostasis, thereby conferring susceptibility to experimentally induced colitis^47^. To address whether *Cdk5rap3* deficiency predisposes susceptibility to inflammatory colitis, we tested IEC-specific *Cdk5rap3* deficient mice in the DSS (dextran sulfate sodium)-induced colitis model. In comparison to wild-type mice, *Cdk5rap3*^∆/∆IEC^ mice exhibited accelerated weight loss (Fig. 4a), deteriorated clinical scores (Fig. 4b), and colon shrinkage (Fig. 4c). In addition, *Cdk5rap3*^∆/∆IEC^ mice possessed severe epithelial damage including significant loss of basal crypts, massive infiltration of immune cells (Fig. 4d), and elevated expression of inflammatory cytokines IL-6, IL-1β and TNFα (Fig. 4e). Altogether, these results demonstrate that *Cdk5rap3*^∆/∆IEC^ mice are more susceptible to DSS-induced colitis.

### *Cdk5rap3* knockout results in defective ufmylation pathway and activation of Unfolded Protein Response (UPR)

In attempt to gain insight into the molecular mechanism of Cdk5rap3’s function, we examined gene expression profiles in wild-type and *Cdk5rap3*^∆/∆IEC^ intestine. Among 2674 DEGs (differentially expressed genes) (cutoff 1.3-fold, *P*_adj_ < 0.05), 1400 genes were upregulated and 1274 genes were downregulated in *Cdk5rap3*^∆/∆IEC^ small intestine (Supplementary Tables S1 and S2, A1-A3 for *Cdk5rap3*^f/f^, and A4-A8 for *Cdk5rap3*^∆/∆IEC^). Consistent with our results described above (Fig. 1h), RNA-seq analysis confirmed down-regulation of Paneth cell-specific defensin genes (Supplementary Table S2 and Supplementary Fig. S3a). Furthermore, RNA-Seq result validated the under-expression of Gfi1 and Sox9 and up-regulation of Olfm4 induced by *Cdk5rap3* deletion (Fig. 2d, Supplementary Tables S1 and S2 and Supplementary Fig. S3b).

**Fig. 5:**
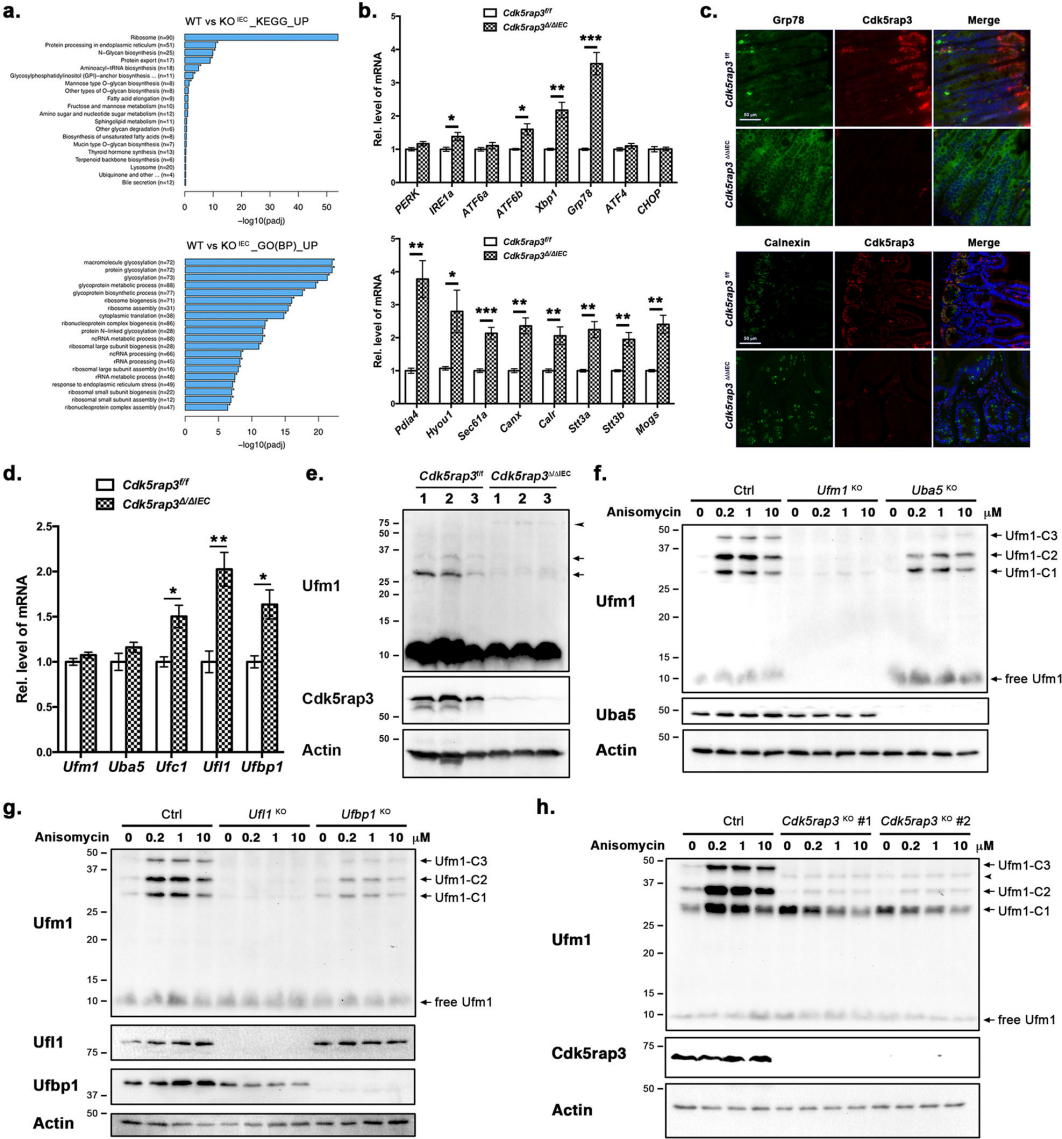
*Cdk5rap3* knockout results in defective ufmylation pathway and activation of Unfolded Protein Response (UPR). **a** GO and KEGG enrichment analysis of up-regulated DEGs in *Cdkrap3*^∆/∆IEC^ intestine. **b** Quantitative RT-PCR confirmation of up-regulated genes that are involved in UPR and ER-related protein glycosylation and folding. *p < 0.05, **p < 0.01, ***p < 0.001 (*n* = 4 mice per genotype). **c** Immunofluorescence staining of Grp78/Bip and Calnexin in ileal section of wild-type and *Cdkrap3*^∆/∆IEC^ mice. **d** Up-regulation of the components of the Ufm1 conjugation system induced by *Cdk5rap3* knockout. Total RNA was isolated from small intestinal scrapes. *p < 0.05, **p < 0.01 (*n* = 4 mice per genotype). **e** The Ufm1 conjugates in wild-type and *Cdk5rap3* KO IECs. Cell lysates of isolated crypts were subjected to immunoblotting of Ufm1. Endogenous of Ufm1 conjugates in wild-type cells were marked by arrows, while a new Ufm1 conjugate in *Cdk5rap3* KO cells was indicated by an arrowhead. **f** UFMylation in wild-type, *Ufm1* KO and *Uba5* KO 293 T cells in the absence and presence of anisomycin. Cells were treated with anisomycin for 1 h. Ufm1-C1, 2, 3 are endogenous Ufm1 conjugates. **g** UFMylation in wild-type, *Ufl1* KO and *Ufbp1* KO 293T cells with and without anisomycin. **h** UFMylation in wild-type and *Cdk5rap3* KO 293T cells with and without anisomycin. A new Ufm1 conjugate in *Cdk5rap3* KO cells was indicated by an arrowhead (around 45 kD).

We then performed Gene Ontology (GO) of Kyoto Encyclopedia of Genes and Genomes (KEGG) enrichment analysis of upregulated DEGs. As shown in Fig. 5a, the top hits included ribosome biogenesis, protein translation, glycosylation and processing in the ER, cellular response to ER stress, and other metabolic pathways such as lipid metabolism (Fig. 5a). Cdk5rap3 was identified as an interacting protein of Ufl1, a Ufm1-specific E3 ligase^25,27^. The Ufm1 conjugation system is a novel ubiquitin-like modification system that consists of Uba5 (Ufm1-specific E1 enzyme), Ufc1 (Ufm1-specific E2 enzyme), and Ufl1/Ufbp1 complex (Ufm1-specific E3 ligase)^48,49^. Previous studies have demonstrated its indispensable role in mouse embryogenesis and erythropoiesis^50–52^. We have recently reported that Ufl1 and Ufbp1 is essential for maintenance of intestinal homeostasis, and knockout of Ufl1 and its co-factor Ufbp1 led to complete ablation of Paneth cells and partial loss of goblet cells^47^. Elevated ER stress and UPR activation has been observed in both *Ufbp1* KO intestine and B cells as well as several UFMylation-deficient cell lines^21,47,53^. Therefore, we examined UPR activation first. As shown in Fig. 5b, *Cdk5rap3* deletion caused significant up-regulation of ER stress sensors IRE1α and ATF6β, but not PERK and ATF6α (Fig. 5b). Moreover, expression of IRE1α downstream effector Xbp-1 and its target Grp78/Bip were also significantly increased (Fig. 5b), indicating that *Cdk5rap3* ablation activates the IRE1α-Xbp-1 branch of the UPR. In contrast, expression of ATF4 and CHOP, two downstream targets of the PERK branch, was not altered by *Cdk5rap3* KO, suggesting that the PERK branch may not be activated in *Cdk5rap3* KO intestine. Furthermore, representative genes in glycosylation (*Stt3a*, *Stt3b*, and *Mogs*), protein folding and chaperones (*Pdia4*, *Hyou1*, *Canx*, and *Calr*), and co-translational translocation (*Sec61a*) were also upregulated in *Cdk5rap3* KO intestine (Fig. 5b). Activation of the UPR in *Cdk5rap3* deficient cells was further confirmed by enhanced immunostaining of Grp78/Bip and Calnexin (Fig. 5c). Taken together, our results suggest that like *Ufl1* and *Ufbp1*, *Cdk5rap3* deficiency may lead to activation of the UPR, especially the IRE1α-Xbp-1 branch.

As an interacting protein of Ufm1-specific E3 Ligase Ufl1, Cdk5rap3 has been shown to modulate the UFMylation pathway^21,22,54^. Interestingly, loss of *Cdk5rap3* in IECs led to elevated expression of other components of the Ufm1 system, including Ufc1 (Ufm1 E2 enzyme), Ufl1 and Ufbp1 (Ufm1 E3 ligase) (Fig. 5d), indicating a possible compensatory response to *Cdk5rap3* deficiency. Knockout of *Cdk5rap3* also altered UFMylation of endogenous targets, as evidenced by a reduction of several endogenous Ufm1 conjugates and appearance of new Ufm1 conjugate (around 80 kD, Fig. 5e). To further explore the impact of Cdk5rap3 on the UFMylation pathway, we examined Ufm1 conjugation in HEK293T cells under various conditions. It has been shown that low concentration of translation elongation inhibitor anisomycin causes ribosome stalling and collision, resulting in increased Ufm1 conjugation of ribosomal protein RPL26^55^. As shown in Fig. 5f, *Ufm1* KO completely abolished basal and anisomycin-induced UFMylation, while *Uba5* KO (Ufm1 E1 ligase) partially blocked UFMylation. Similarly, UFMylation was almost completely eliminated by *Ufl1* KO and attenuated by *Ufbp1* deletion (Fig. 5g). In comparison to parental cells, *Cdk5rap3* KO attenuated basal and anisomycin-induced multi-UFMylation (Ufm1 conjugates C2 and C3) but enhanced basal mono-UFMylation (Ufm1-C1) (Fig. 5h). Mono-UFMylated form in *Cdk5rap3* KO cells was slightly reduced by anisomycin treatment, possibly due to anisomycin-induced multi-UFMylation of this mono-form (Fig. 5h). Collectively, our data strongly suggest that Cdk5rap3 plays a pivotal role in the UFMylation pathway, and alteration of UFMylation caused by *Cdk5rap3* deficiency may lead to activation of UPR and loss of Paneth cells.

## Discussion

In this report, we present the genetic evidence to demonstrate the pivotal role of Cdk5rap3 protein in Paneth cell development and intestinal homeostasis. IEC-specific deletion of *Cdk5rap3* caused nearly complete loss of Paneth cells (Fig. 1). Gene expression analysis revealed that *Cdk5rap3* deficiency resulted in under-expression of key TFs that are important for Paneth cell lineage specification (Fig. 2 and Supplementary Table S2), suggesting a crucial role of Cdk5rap3 in cell fate decision. Moreover, Paneth cell-specific *Cdk5rap3* knockout caused partial loss of Paneth cells, and TAM-induced acute deletion of *Cdk5rap3* led to abnormal Paneth cells and subcellular abnormalities (Fig. 3), indicating its important role in mature Paneth cells. Consequently, *Cdk5rap3* deficient mice manifested increased susceptibility to DSS-induced colitis (Fig. 4). Taken together, our results establish Cdk5rap3 as an important regulator of Paneth cell development.

The intestinal epithelium is one of the fastest renewing tissues, and the renewal process is driven by intestinal stem cell under stringent control of multiple signaling pathways such as Wnt, Notch and BMP pathways^56^. Under physiological condition, the Wnt pathway is the dominant force to drive proliferation of Lgr5^+^ intestinal stem cells. Aberrant activation of this pathway results in intestinal hyperplasia and colorectal cancers, whereas its inhibition leads to loss of crypts and altered lineage development^57,58^. In comparison, the Notch pathway is not only essential for maintenance of stem cell compartment, but also controls absorptive versus secretory cell fate specification. Inhibition of Notch signaling drives stem cells towards secretory lineages^59^. Paneth cell development is tightly controlled by both Wnt and Notch pathways and their downstream transcription factors^60^. The combination of Wnt activation and Notch inhibition promotes Lgr5^+^ ISCs to differentiate to Paneth cells in organoid culture^61^. Deletion of *Lgr4*, a positive regulator of Wnt signaling, significantly impaired Paneth cell formation^62^. Math1/Atoh1 is a master regulator of secretory cells and essential for development of all secretory lineages, including both exocrine (Paneth and goblet) and enteroendocrine lineages^35,37^. Sox-9, a Wnt/β-catenin target, is essential for Paneth cell fate decision and differentiation^38,39^. Gfi1 is important for fate decision of exocrine cell lineage, while Sox9 is specifically required for differentiation of Paneth cells^36,38,39^. Interestingly, β-catenin activity is required for Paneth cell maturation^63^. Yet, how these signaling pathways and transcription factors coordinate to control differentiation, maturation and plasticity of Paneth cells remains poorly understood.

Interestingly, our result shows that Cdk5rap3 is critical for both fate decision and development of Paneth cells, a feature that distinguishes Cdk5rap3 from other important regulators of Paneth cell development. On the one hand, Cdk5rap3 is critical for Paneth cell specification. *Cdk5rap3* knockout led to down-regulation of Gfi1 and Sox9 (Fig. 2a), and *Cdk5rap3* deficient intestine was similar to the ones of *Gfi1* and *Sox9* KO mice. Yet there are several phenotypic differences among these KO mouse models. *Cdk5rap3* KO mice exhibited neither significant increase of enteroendocrine cells as manifested in *Gfi1* KO mice nor apparent crypt enlargement as observed in *Sox9* KO mice^36,38,39^. On the other hand, Cdk5rap3 is also important for mature Paneth cells, and Paneth cell-specific deletion led to Paneth cell loss (Fig. 3a–c). Taken together, our data strongly suggest that Cdk5rap3 represents a novel and unique regulator of Paneth cell biology.

What is the molecular mechanism underlying Cdk5rap3’s function? One of possible mechanisms is its role in the UFMylation pathway. The Ufm1 conjugation system is involved in multiple signaling pathways and cellular processes, including transcriptional regulation^64^, p53 stability^65^, DNA damage response^66,67^, autophagy^68,69^ and UPR^70,71^. Notably, the Ufm1-specific E3 ligase consisting of Ufl1/Ufbp1 is highly expressed in intestinal exocrine cells and vital for their survival and function^47^. IEC-specific knockout of either *Ufl1* and *Ufbp1* leads to almost complete loss of Paneth cells and partial loss of goblet cells^47^. Cdk5rap3 is an interacting partner of Ufl1 and Ufbp1^25–27^. Therefore, it is plausible that Cdk5rap3 may affect Paneth cell development and function through modulating the UFMylation pathway. Indeed, its deficiency alters basal and anisomycin-induced UFMylation of endogenous targets (Fig. 5), a result that is consistent with previous observations^21,22^. Moreover, similar to Ufl1 and Ufbp1, Cdk5rap3 deletion causes preferentially activation of the IRE1α branch of UPR (Fig. 5). Although how UFMylation deficiency increases ER stress remains elusive, elevated ER stress and UPR activation may affect many aspects of intestinal homeostasis and physiology, including reduced stemness of ISC and impaired development and survival of exocrine cells^72^. Further studies will be conducted to explore the underlying mechanism of cross-talk between the ER and the UFMylation pathway and its impact on Paneth cell development.

Despite phenotypic similarities shared by *Cdk5rap3* and *Ufl1/Ufbp1* KO mice, there are also several notable differences. *Cdkr5rap3* KO embryos die much earlier than *Ufl1* and *Ufbp1* null embryos^52^. Additionally, no severe anemia was observed in adult *Cdk5rap3* conditional KO mice (our unpublished observation), suggesting that unlike other components of the Ufm1 system, Cdk5rap3 is not essential for red blood cell development. Interestingly, Cdk5rap3 appears to have a distinct effect on UFMylation. One of the principal Ufm1 targets is RPL26, a ribosomal protein whose UFMylation is enhanced by ribosome stalling^21,55^. Ufm1-C1 and C2 conjugates represent mono- and di-UFMylation of RPL26 (Fig. 5)^21,55^. As shown in Fig. 5f and g, *Ufl1* KO nearly abolishes RPL26 UFMylation, while *Uba5* or *Ufbp1* KO significantly attenuates its UFMylation. Interestingly, *Cdk5rap3* KO cells exhibited a distinct UFMylation pattern. Specifically, multi-UFMylation was dramatically diminished by *Cdk5rap3* KO while basal RPL26 mono-UFMylation is substantially increased (Fig. 5h). Additional Ufm1 conjugate was also observed in *Cdk5rap3* KO cells (Fig. 5h). It remains to be determined whether the differential influence on UFMylation contributes to the phenotypic difference of *Cdk5rap3* and *Ufl1/Ufbp1* KO mice.

In addition to the UFMylation pathway, Cdk5rap3 has been implicated in other signaling pathways. Several studies suggest that Cdk5rap3 may directly modulate transcription by interacting with TFs such as RelA and CBP/p300^11,12^. Therefore, it is plausible that Cdk5rap3 may directly regulate expression of key TFs such as Gfi1 and Sox9 to control lineage allocation. Alternatively, cytosolic Cdk5rap3 may modulate WNT signaling to affect intestinal development. Previous studies have indicated that Cdk5rap3 is a negative regulator of WNT signaling by stabilizing GSK-3β^15,16^. More studies will be conducted to determine whether *Cdk5rap3* deficiency results in altered output of WNT signaling and whether Cdk5rap3 is directly involved in regulation of WNT signaling in the intestine. Furthermore, *Cdk5rap3* deficiency also affected cell cycle progression of proliferating IECs (Fig. 1j) and ribosome biogenesis (Fig. 5a), indicating its involvement in multiple cellular processes. Given a long list of its potential interacting proteins, further mechanistic investigations will establish whether Cdk5rap3 acts in a unified molecular mechanism or it is a truly multi-faceted protein with pleotropic functions in different type of cells and tissues.

## Materials and methods

### Generation of Cdk5rap3 knockout mice and genotyping protocol

ES cell clone HEPD0516_2_A06 (JM8.N4 with C57BL/6N background) containing trapped *Cdk5rap3* allele was purchased from the EUCOMM (European Conditional Mouse Mutagenesis) team (supplemental Fig. 1). The ES cells were injected into the blastocysts of C57BL/6 mice (Northwestern University Transgenic and Targeted Mutagenesis Laboratory). Chimeric mice were crossed with B6(Cg)-Tyr^*C-2J*^/J albino mice, and heterozygous offspring with germ-line transmission were confirmed by genotyping.

To generate conditional KO mice, we crossed *Cdk5rap3*^Trap-F/+^ mice with FLPo deleter mice (stock # 011065, The Jackson Laboratory, Bar harbor, ME) to remove the gene trap cassette. The *Cdk5rap3* floxed mice were crossed with various Cre strain to generate tissue and cell-type specific KO mice. For tamoxifen-induced whole body KO, CAG-CreERT2 mice (stock# 004453, The Jackson Laboratory) was crossed with *Cdk5rap3* floxed mice, and Cdk5rap3 deletion was induced by tamoxifen injection. Tamoxifen (20 mg/ml in corn oil, Sigma, St. Louis, MO) was administrated by 5-day IP injection with an approximate dose of 75 mg tamoxifen/kg body weight. For IEC-specific KO, *Cdk5rap3* floxed mice were crossed with Villin-Cre transgenic mouse that was originally from Dr. Sylvie Robine’s laboratory^73^. Mice were housed in the animal facility of Augusta University, and all animal procedures were approved by AU IACUC. All mice used in this study were at the age of 8–12 weeks, and randomly chosen for the experiments.

The following primers were used for PCR genotyping of *Cdk5rap3 trapped allele*: F1 (CACAACGGGTTCTTCTGTTAGTCC), R1 (GATTGGCAGGAGATCGTAAGCCTG) and R2: (GCAGTACGCACTACCTCCCCAAGG). A 35-cycle (92 °C, 45 s, 62 °C, 45 s, 72 °C 45 s), 260 bp PCR product is for wild-type allele, while 425 bp is for trapped allele. For Cdk5rap3 floxed allele, two primers are used: P1 (TAG CTC GGG GCT CAG ACG CTC TGA) and P2 (TTA TCT GCT CTT CCC GCT AGA ATA). The floxed allele generates a 356 bp PCR product while wild-type allele gives 330 bp product. Genotyping of Cre-ERT2 mice was performed according to the standard protocol of the Jackson Laboratory, while Villin-Cre and D6-Cre mice were genotyped as previously described^41^.

### Isolation and in vitro organoid culture of intestinal crypts

Crypts were isolated from the ileum of small intestine according to Sato et al. with minor modifications^74^. Briefly, the ileum of small intestine was harvested and opened lengthwise, and then washed multiple times with cold PBS. After removal of the villi with a cover glass, the intestine was cut into 2–3 large pieces, and then washed with cold PBS (10–20 ml) for more than ten times. Subsequently the fragments were incubated in 25 ml of 2 mM EDTA on ice for 15 min with a gentle shaking. After removal of the supernatant, the tissue was washed with cold PBS and then pipetted up and down 3–5 times. Released crypts were passed through a 70 μM cell strainer and collected by 3-min centrifugation at 100 × *g*. Isolated crypts were counted and resuspended in Growth Factor Reduced Matrigel (Corning Life sciences, Corning, NY) and cultured in the complete medium: Advanced DMEM/F12 supplemented with 1× B27, 1× N2 (Thermo Fisher Scientific, Waltham, MA), 1 mM N-acetyle-L-cysteine, HEPES (10 mM, pH 7.4), murine EGF (50 ng/ml, PeproTech, Rocky Hill, NJ), murine Noggin (100 ng/ml, PeproTech), 1/10th volume of R-Spondin-1 conditional medium (Trevigen, Gaitherburg, MD), 1 × GlutaMax, 1 × Penicillin/Streptomycin (ThermoFisher Scientific) and 100 μg/ml Normocin (InvivoGen, San Diego, CA),

### RNA-seq, differential, and enrichment analysis

The cells were scraped off from ileal section of small intestine with slide glass. Total RNAs from three wild-type and four *Cdk5rap3* KO mice were isolated from the scrapes with TRIzol (ThermoFisher Scientific) and Zymo Direct-zol RNA Miniprep Plus kit (R2072, Zymo Research, Irvine, CA). RNA-seq and analyses were conducted by Novogene (Beijing, China). Raw data were obtained by Illumina platform. After data filtering, the genes were mapped to the genome with STAR software, and quantification was determined by HTSeq software. DESeq2 was used for differential analysis, and ClusterProfiler was used for enrichment analysis (GO, KEGG and Reactome). Gene Set Enrichment Analysis was performed online at https://www.gsea-msigdb.org/gsea/index.jsp.

### Histology, immunohistochemistry, immunofluorescent staining, and immunoblotting

HE and PAS/Alcian blue staining was performed by the Histology core of Augusta University, while TEM was conducted by the EM core of Augusta University according to the standard procedures. Immunohistochemistry, immunofluorescent staining, and immunoblotting were performed as described previously^45^. Bright field and Epifluorescence images were obtained using Zeiss Observer D1 with AxioVision 4.8 software (Carl Zeiss Microscopy GmbH, Jena, Germany) and Keyence BZ-X700 fluorescent microscope with its corresponding software (Keyence America, Itasca, IL, USA). All histopathological analysis and quantification were performed blindly by lab personnel who had no prior information on the genotypes of animals and tissues.

The antibodies used in this study included: Cdk5rap3 rat polyclonal antibody (Li lab), Uba5 (Li Lab), Ufl1 (Li lab), Ufbp1 (21445-1-AP, Proteintech, Rosemont, IL), phospho-Histone H3 (ser10) (#9706, Cell Signaling), PCNA (#2586, Cell Signaling), Olfm4 (#39141, Cell Signaling), Calnexin (#2679, Cell Signaling), Ufm1 (Ab109305, Abcam), Lysozyme (A0099, Agilent Dako, Santa Clara, CA), Grp78/Bip (#3177, Cell Signaling), Chromogranin A (Ab15160, Abcam, Cambridge, MA), and β-Actin (#3700, Cell Signaling). All affinity-purified and species-specific HRP- and fluorophore-conjugated secondary antibodies were obtained from Jackson ImmunoResearch (West Grove, PA).

### Generation of CRISPR/cas-9-mediated knockout cell lines

The plasmids containing sgRNAs were constructed by cloning sgRNA-containing oligos into pLentiCRISPR V2 vector (Addgene # 52961). Lentiviral particles expressing sgRNA and cas-9 were prepared by standard 293T cell transfection, and then used for infection of parental 293T cells. Knockout cell clones were isolated by puromycin selection, limiting dilution, and immunoblotting. Sequences of sgRNAs were listed below:

**Table:** 

Gene (human)	sgRNA
Cdk5rap3 sgRNA-1	GTTGACATTCCGAACCAGG
Cdk5rap3 sgRNA-2	GATTATAGCTCTGTATGAGA
Ufm1 sgRNA-1	TCACGCTGACGTCGGACCCA
Ufm1 sgRNA-2	CTTTAAGATCACGCTGACGT
Uba5 sgRNA-1	TCCCGAGGAGCGGCGACGGA
Uba5 sgRNA-2	GCTGGAGCGGGAACTTGCCC
Ufl1 sgRNA-1	CCAGCGGGCGCAGTTCGCCG
Ufl1 sgRNA-2	GGAAGAGATTAGGCGGTTGG
Ufbp1 sgRNA-1	GTAGCGGCGGCTCTGCTAGT

### Quantitative real-time PCR

Total RNA was isolated with TRIzol (ThermoFisher Scientific) and Zymo Direct-zol RNA Miniprep Plus kit (R2072, Zymo Research, Irvine, CA), and then reversely transcribed with High-Capacity cDNA Reverse Transcription kit according to the manufacturer’s instruction (ThermoFisher Scientific). Quantitative RT-PCR was performed using the iTaq Universal SYBR Green Supermix kit (BIO-RAD, Hercules, CA) with 40 cycles of 95 °C for 15 s and 60 °C for 1 min on StepOnePlus Real-Time PCR System (ThermoFisher Scientific). The results were analyzed by StepOne Software (Version 2.1, Life Technologies). Relative expression of each transcript was normalized to murine beta-actin by using the 2^(-delta delta Ct) method. The following is the list of primers used in this study:

**Table:** 

Gene (mouse)	Forward primer	Reverse primer
Actin	GACCTCTATGCCAACACAGT	AGTACTTGCGCTCAGGAGGA
Cdk5rap3 (exon6-12)	TGGAAGCTGTTTCCGACTCT	AGCCTCAGTTCCTGTCTCCA
Lysozyme 2	ATGGAATGGCTGGCTACTATGG	ACCAGTATCGGCTATTGATCTGA
Defcr1	AAGAGACTAAAACTGAGGAGCAGC	CGACAGCAGAGCGTGTA
Defcr5	AGGCTGATCCTATCCACAAAACAG	TGAAGAGCAGACCCTTCTTGGC
Intestinal ALP	CACAGCTTACCTGGCACTGA	GGTCTCTGACGACAGGGGTA
Chromogranin A	CCAATACCCAATCACCAACC	TTGTAGCCTGCATGGAAGTG
Mucin 2	GCCTGTTTGATAGCTGCTATGTGCC	GTTCCGCCAGTCAATGCAGACAC
Lgr5	ACCCGCCAGTCTCCTACATC	GCATCTAGGCGCAGGGATTG
Olfm4	TGGCCCTTGGAAGCTGTAGT	ACCTCCTTGGCCATAGCGAA
Math1	GAGTGGGCTGAGGTAAAAGAGT	GGTCGGTGCTATCCAGGAG
Hes1	CCAGCCAGTGTCAACACGA	AATGCCGGGAGCTATCTTTCT
Gfi1	AGGAACGCAGCTTTGACTGT	CCTGTGTGGATGAAGGTGTG
Sox9	GAGGAAGTCGGTGAAGAACG	CCCTCTCGCTTCAGATCAAC
Klf4	CTGAACAGCAGGGACTGTCA	GTGTGGGTGGCTGTTCTTTT
Elf3	GCATGTCCTTCCAAGAGAGC	ACATCACTTCCACCGGAGTC
IL-6	TAGTCCTTCCTACCCCAATTTCC	TTGGTCCTTAGCCACTCCTTC
IL-1β	GCCCATCCTCTGTGACTCAT	AGGCCACAGGTATTTTGTCG
TNFα	CCGATGGGTTGTACCTTGTC	TGGAAGACTCCTCCCAGGTA
Perk	GAAACGGCTTTCAGTTGAGC	CTGGCCATATCCACCAGAGT
Ire1α	CAGATCTGCGCAAATTCAGA	CTCCATGGCTTGGTAGGTGT
Atf6α	AGCCGACTGTGGTTCAACTT	CCCATACTTCTGGTGGCACT
Atf6β	TTTGACAGCAGCTCTCTGGA	CATCTTCACATGCAGCACCT
Xbp1	ACACGCTTGGGAATGGACAC	CCATGGGAAGATGTTCTGGG
Grp78/Bip	ACTTGGGGACCACCTATTCCT	ATCGCCAATCAGACGCTCC
Atf4	GAAACCTCATGGGTTCTCCA	AGCTCATCTGGCATGGTTTC
Chop	GCATGAAGGAGAAGGAGCAG	ATGGTGCTGGGTACACTTCC
Pdia4	TGCTGACACACCCTGAGAAG	TGCTGTACCGCTTAGCATCA
Hyou1	GCCCACTTTAACCTGGATGA	GGCTCTCCTCTTCCTCCTGT
Sec61α	CTATTTCCAGGGCTTCCGAGT	AGGTGTTGTACTGGCCTCGGT
Canx	ACAAGAGCGATTGGATGGAA	TGCTTGTGAATGGAGCAGTC
Calr	AGGCTCCTTGGAGGATGATT	TCCCACTCTCCATCCATCTC
Stt3a	TATCTCCCTGGTTGGCTTTG	TGGTGCTCAGAAACAGATGC
Stt3b	TGGAGGACAGCAGTGATGAG	AAGGACCACACTTGGACTGG
Mogs	GGACCTAGCTTTGCCTACCC	TTCAGTCTCCCCAAGCTGTT
Ufm1	CCGTTCACAGCAGTGCTAAA	CAGCTTCCAACTCGGTCTCT
Uba5	CAAGCTATGTTCACGGCAGA	AGTTGTTTTGCCCACCACTC
Ufc1	AACTGCACTTCCGCAGTTTT	CTCCAGTCGGAACCAATCAT
Ufl1	AGCAAACAGGCCTCAACTGT	TTTCTGGTGCATCAGCTCAC
Ufbp1	GAAGCCAGCAGAAGTTCACC	GAAGCCGTTCCTCTTCCTTC

### Statistical analysis

All statistical analyses were performed using Graph Prism 7 software. *P* values were determined by unpaired *t*-tests between two set of data. A *p* value < 0.05 was considered to be significant.

### TUNEL staining

TUNEL staining was performed using in situ cell death detection kit (TMR Red, Roche, Basel, Switzerland) according to the manufacturer’s instruction.

## Supplementary information

Supplementary figure

Supplementary table 1

Supplementary table 2
