# Supplementary material for: Cdk5rap3 is essential for intestinal Paneth cell development and maintenance
Source: Cell Death Dis. 2021 Jan 27;12(1):131. doi: 10.1038/s41419-021-03401-8 (PMC7841144; doi:10.1038/s41419-021-03401-8)
Supplement: Supplementary file 1 — Supplementary figure [file 41419_2021_3401_MOESM1_ESM.docx]

Supplemental Fig. 1


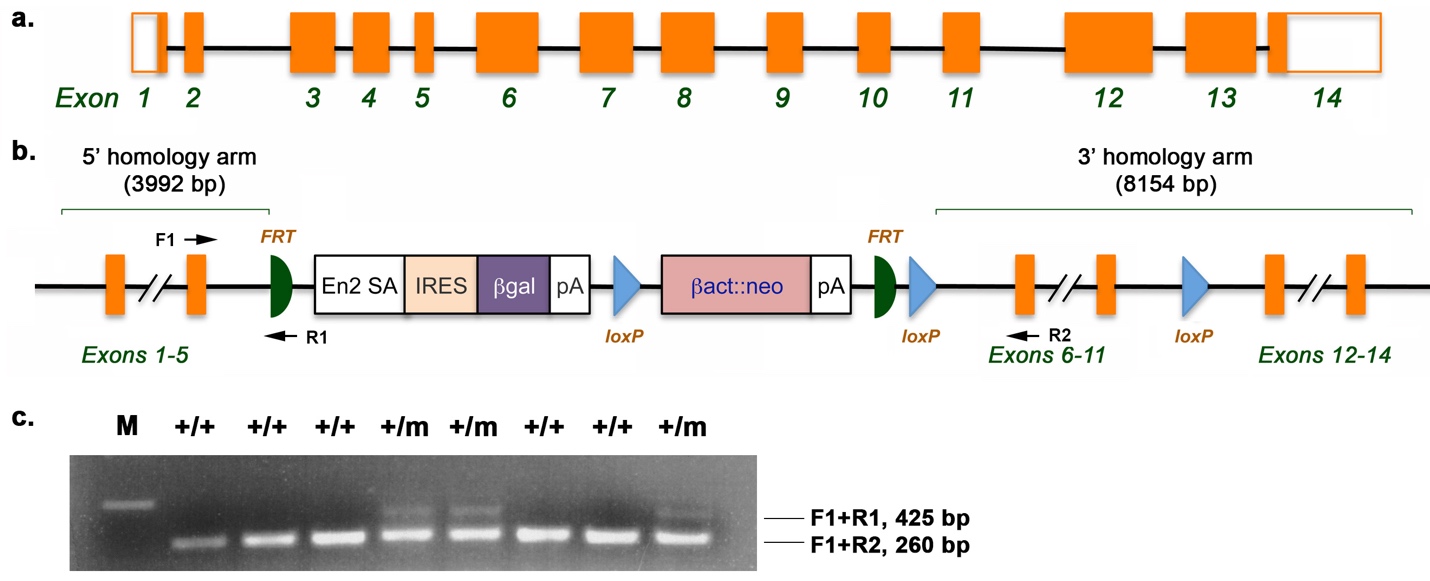


sFig. 1: **a.** The gene structure of mouse *Cdk5rap3* in chr.4. **b.** The targeting vector of *Cdk5rap3* allele. **c.** The genotype of pups from crossing of heterozygous mice with knockout allele. We have examined more than 10 litters and failed to obtain pups with homozygous knockout allele (m/m) of *Cdk5rap3***.**

Supplemental Fig. 2


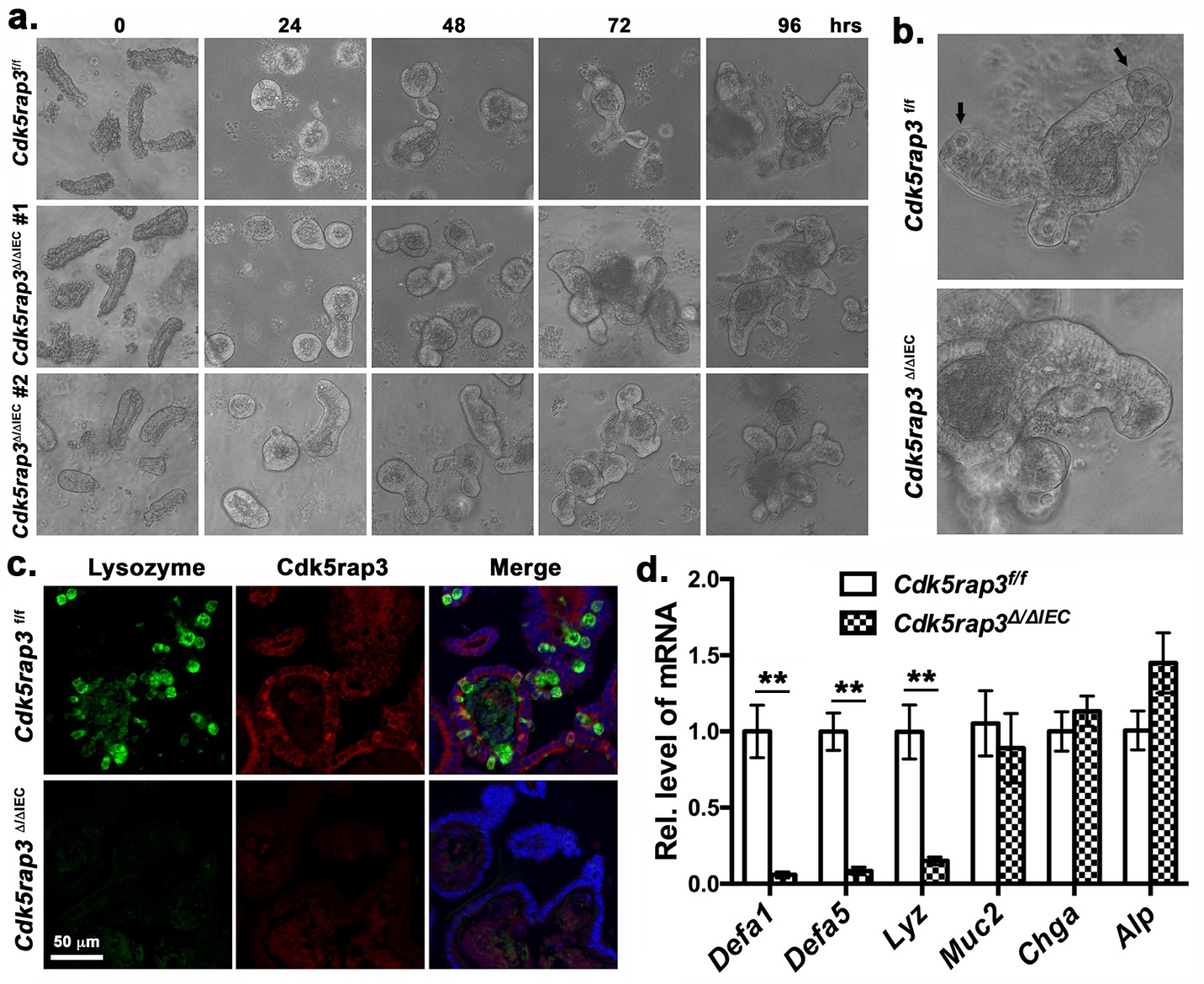


sFig. 2 **a.** Time course of organoid culture of wt and *Cdk5rap3* KO crypts. **b.** Wt and *Cdk5rap3* KO organoids. Paneth cells were marked by black arrows. **c.** Lysozyme staining of sections of wt and *Cdk5rap3* KO organoids. **d.** Quantitative RT-PCR analysis of cell type-specific gene expression**.** *p* values were determined by unpaired *t*-tests. ****** p < 0.01 (n=4).

Supplemental Figure 3:


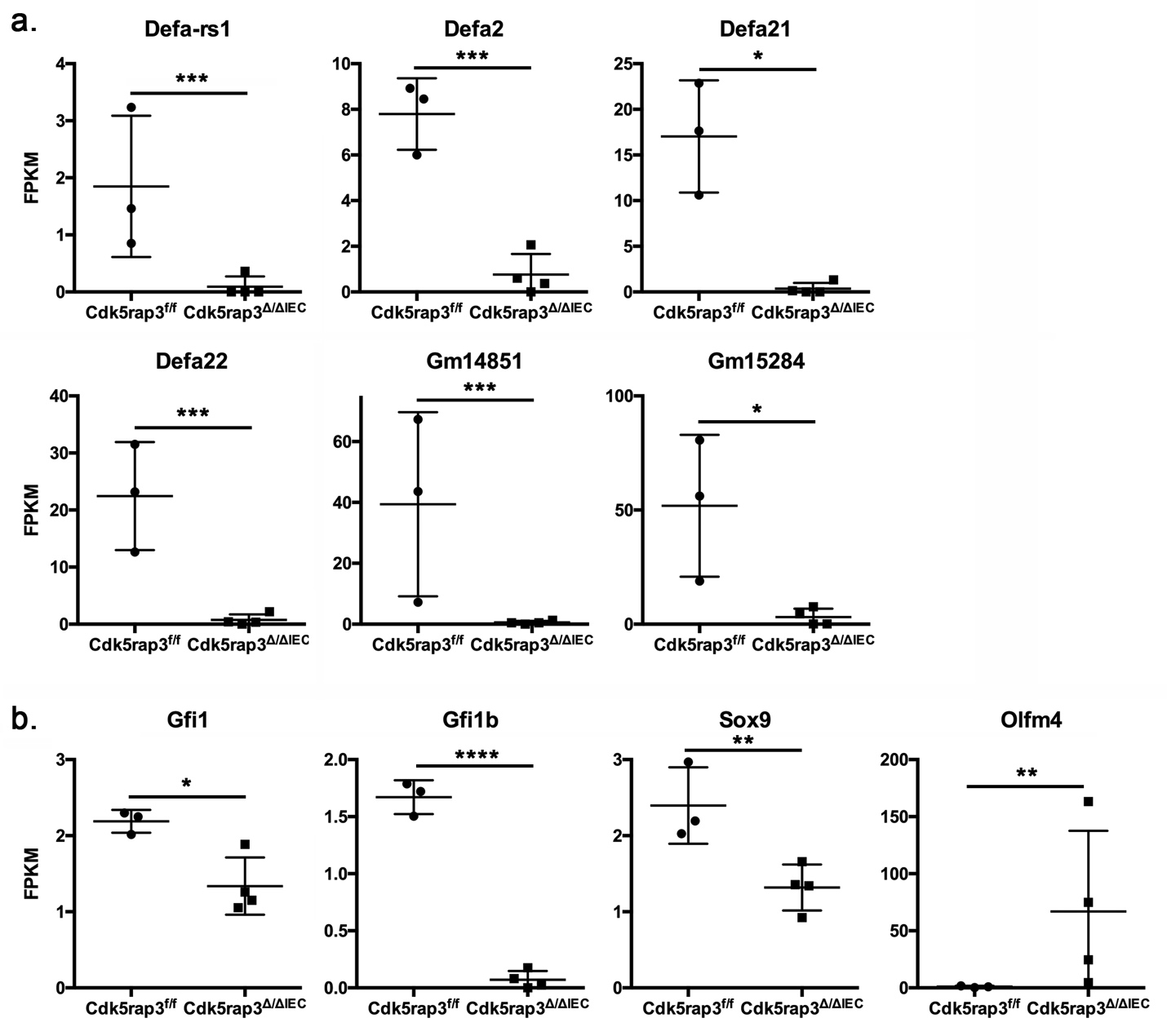


sFig.3. Expression of representative genes in wild-type and *Cdk5rap3*^∆/∆IEC^ intestine. **a.** Expression of various Paneth cell-specific defensin genes. **b.** Expression of Gfi1, Sox9 and Olfm1 genes in wild-type and *Cdk5rap3*^∆/∆IEC^ intestine. FPKM (Fragments Per kilobase of transcripts per Million) values were calculated from RNA-seq counts (see supplemental tables 1 and 2), and Padj values were calculated accordingly. * Padj < 0.05; ** Padj < 0.01; *** Padj < 0.001; and **** Padj < 0.0001.
